# How do life course events affect the accumulation of digital literacy?: Based on qualitative research of 16 medical university teachers of China

**DOI:** 10.1097/MD.0000000000038755

**Published:** 2024-07-05

**Authors:** Wei Li, Shaojie Yu, Mingling Wang, Xuening Li, Guangbin Ma, Xuehong Ju, Chunguang Ling

**Affiliations:** a Department of Graduate, Shandong Second Medical University, Weifang, China; b School of Psychology, Shandong Second University, Weifang, China.

**Keywords:** cumulative advantage, digital literacy, life course theory, medical university teachers

## Abstract

The purpose of this article is to examine the level of the accumulation of digital literacy in medical university of China. In this study, we argue that positive life events facilitate the formation of digital literacy. We review the development of research of life course and digital literacy since the 2010. From the perspective of life course theory, this article examines the significant impact of social changes in information technology on the accumulation of digital literacy of 16 medical university teachers, and understands the life course of medical university teachers as a sequence composed of multiple life events. The results show that the accumulation of medical university teachers’ digital Literacy includes 4 types: linear accumulation, multi drive accumulation, parallel accumulation, and leading accumulation, of which multi drive accumulation and leading accumulation are the most conducive to the formation of medical university teachers’ digital literacy. In addition, our findings reveal that subjective initiative plays an important role in the accumulation of medical university teachers’ digital literacy. The accumulation of digital literacy is a dynamic and systematic process of the accumulation of individual life events of medical university teachers. This paper also discusses the relationship between order of life events and career outcomes.

## 1. Introduction

Medical universities play a leading role in higher education construction of China. Medical university teachers are the “living soul” of higher education. But the digital literacy of medical university teachers can hardly meet the increasingly diversified needs of digital society.^[1,2]^ The government popularizes information technology in medical university, and implements “Internet + higher education” in a planned way. It puts forward higher requirements for medical university teachers to strengthen digital literacy. The Ministry of Education of China issued 《The Opinions on Strengthening the Construction of University Teachers in New Era》 in 2020, and government implements the 2.0 project to improve the application ability of digital literacy for medical university teachers.^[3]^ The law encourages qualified regions to explore first, and promotes the deep integration of information technology and higher education. Medical university teachers should have enough digital literacy to practice the integration of information technology and teaching,^[4]^ and face the new role in the digital era.

The accumulation of digital literacy plays an important role in the career development of medical university teachers, but there is still a lack of special research. The theory of life course is a new perspective to study the accumulation of medical university teachers’ digital literacy.^[5]^ Based on the above analysis, this research summarizes the types and characteristics of medical university teachers’ digital literacy accumulation in China, and lists typical cases. This paper aims to explore the rule of medical university teachers’ digital literacy accumulation, and expands the new perspective of medical university teachers’ digital literacy research.

## 2. Literature review

The course belongs to the concept of the sociological research field, and life course theory is an important area of sociological research. American educational researchers often use life course theory as a research perspective or a research path, and sometimes as an analytical entry point to an educational problem.^[6]^ Some researches have focused on the differences in educational attainment brought about by differences in life trajectories, which in turn explain the reasons for the vastly different careers, such as 《Educational Transitions, Trajectories, and Pathways》 《Education Pathways》 《The life course as an observational medium in the education system》 《Education, training and social inequalities across the life course》. Thus, life course theory can serve as both a theoretical basis for cumulative research on teaching literacy, and can be considered an important research topic in the field of educational research.^[7]^

The life course theory paradigm draws on the findings of life-history studies, peer group and age stratification theories, and cultural and generational models to provide researchers with a worldview and methodology for analyzing and solving problems.^[8]^ As urbanization accelerated in the United States in the 1920s and large numbers of medical university people entered the cities, the Chicago School of Thought began to focus on social issues such as immigration, youth transgression, crime, and family marriage. W.I. Thomas 《The Polish peasant in Europe and America》 and G.H. Elder 《Children of the Great Depression》 have studied the life trajectories of immigrants using life history and other methods.^[9]^ The core idea of life course theory is to consider individual life courses as a product of social development and social structure, and trajectory and change are the 2 core concepts of the theory. Later researchers applied the theory to the field of education and studied the trajectory of receiving education as a dynamic tracking research of the life chances of individuals. Brooks argues that factors such as economic conditions, type of employment, learning experience, and digital literacy have a significant impact on adults’ attitudes toward lifelong learning, and that information context can better reflect lifelong learning attitudes.^[10]^ Yung noted that social capital and digital literacy have a positive impact on attitudes toward lifelong learning, that is, the higher the social capital and digital literacy, the higher the attitudes toward lifelong learning.^[11]^ On the other hand, Seamans argues that digital literacy and social capital have a positive impact on the predictive explanatory power of attitudes toward lifelong learning.^[12]^ Unlike sociological research, life course theory research in the field of education analyzes educated individuals or groups and aims to provide answers to educational phenomena and educational questions.^[13]^

## 3. Research design

### 3.1. Sample

The research adopts a combination of convenient sampling and purposeful sampling. Sixteen teachers of different genders and ages were selected from 16 medical universities in 5 cities of Shandong Province as the research objects, as shown in Table 1. The research objects can skillfully use network information technology to carry out teaching activities, and have rich digital literacy accumulation. Compared with other teachers, the research and investigation of the life course of these research objects is more conducive to the in-depth excavation of their accumulated advantages in digital literacy in the process of life growth, and plays a reference role in improving the digital literacy of medical university teachers.

**Table 1:** Sample formation.

Number	Gender	Age	Teach subject
Ms. Zhao	Female	38	Nursing
Mr. Qian	Male	36	Oncology
Ms. Sun	Female	36	Bioengineering
Mr. Li	Female	51	Gastroenterology
Mr Zhou	Male	50	Infectious diseases
Ms. Wu	Female	45	Dermatology
Mr. Zheng	Male	33	Emergency medicine
Ms. Wang	Female	36	Imaging medicine
Mr. Feng	Male	35	Pharmacy
Mr. Chen	Male	48	Anesthesiology
Ms. Chu	Female	44	Urology
Mr. Wei	Male	53	Neurosurgery
Ms. Jiang	Male	45	Clinical diagnosis
Mr. Shen	Male	54	Geriatrics
Ms. Han	Female	47	Public health
Ms. Yang	Female	36	Basic medicine

### 3.2. Methods

The life history school of thought focuses on the activities and events in the life course of the subject in its approach and has developed historical concepts such as age, time, event matrix, and retrospective life calendar.^[14]^ The theoretical origin of the life course doctrine is based on the incorporation of the school of life-history studies, which provides a theoretical basis and research perspective for the research of the relationship between individual life changes and the field of the times in which they live.^[15]^

The research team conducted an in-depth research and survey from April 2022 to January 2023 on the 16 samples. First, a semi-structured interview research method was adopted to grasp first-hand research materials from self-learning, training, informal learning, and life in the course. We focused on depth analysis of important life events such as self-learning IT knowledge, participation in teaching, part-time of academic, and job changing. Second, we used nonparticipant observational research methods to enter the real-life and work situations of participant, and had a direct perceptual understanding. The method provided a concrete argumentative basis for the subsequent research. This research combines the advantages of the 2 research methods above, and places the growth experience of the participants in the social context of the rapid development of information technology in the digital era. The method is conducive to a comprehensive and systematic sorting out of the growth trajectory of participant. We identify the relevant cumulative advantages, and clarify the reform digital literacy of medical university teachers.

## 4. Results

### 4.1. Four types of accumulation

Life course theory is widely used in the acquisition of education, especially the change from one level of education to another.^[16]^ The individual life course of medical teachers exhibits different types and characteristics depending on the length of the life cycle, the width of the life span, and the abundance of the life history. Cumulative theory in life course research considers time and space as 2 important concepts that cover the whole course of individual life: first, time often appears in combination with age in social anthropological research, Time not only represents a point in the life cycle and a historical marker, but also a subjective understanding of the temporality of life. Placing individual age in the social context and peer cohort, we can discover the social roots of individual life changes and transitions.^[17]^ Second, events are the key and core of life course space research. The accumulation and superposition of different events (positive events i.e., cumulative advantages and negative events i.e., cumulative disadvantages) at a fixed point in time will have different effects on the individual life course.^[18]^

Therefore, this research classifies the accumulation of digital literacy of participant into innate accumulation, continuous accumulation and social accumulation. Innate accumulation includes individual cognition, professional background, and academic level. Continuous accumulation includes self-learning, school, and online platform training. Social accumulation includes part-time social work and school employment. According to the above analysis, the types of digital literacy accumulation of participant can be divided into 4 types: linear accumulation, multi-driven accumulation, parallel accumulation, and leading accumulation.

#### 4.1.1. Linear accumulation

In this type, the role of social accumulation on the digital literacy of medical university teachers was not significant, and none of the research participants had taken up part-time social and school-related positions to increase the opportunities to improve digital literacy through social resources. Due to the relatively long time, the innate accumulation of professional background, education level, prior digital literacy accumulation, and other factors can not directly play a role, only through individual learning, school or network platform training to reflect the advantages of professional background. There are even isolated cases in which continuation accumulation works alone. For example, some participants have no training experience in computer technology and network information technology, and their professional backgrounds in clinical medicine, basic medicine, nursing are far from digital literacy. There are also participants who have no accumulation of digital literacy, no interest in digital literacy, and no need for actual teaching work before and at the beginning of their teaching careers.

**Figure 1.**
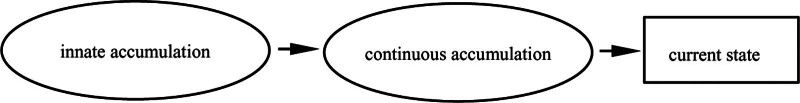
Linear accumulation model.

The basic characteristics of linear accumulation: the effect of innate accumulation is relatively weak, and it is difficult to play a key role in improving the digital literacy of medical university teachers directly and strongly, but innate accumulation can trigger the occurrence of positive life events and make the continuation accumulation play a significant role, thus playing a certain positive role in improving digital literacy of medical university teachers, as shown in Figure 1.

*Case 1: Mr. Chen graduated from a medical university of Shandong Province with an undergraduate degree. Although he took the course of 《Fundamentals of Computer》， he did not know about digital literacy due to the lack of popularity of his major and computer technology. Three years later, he was promoted to a clinical medicine major in a medical college in this province. At the age of 22, Mr. Chen returned to his hometown’s university middle school to teach Chinese Language. He received the training of 《Computer Operation》 during his induction training. In 2000, PPT was popular in teaching. Mr. Chen often searched literature online for teaching preparation. He bought a lot of books about information technology, and learned PPT skill in his spare time. In 2014, the education department of government organized a training class to improve the information teaching ability of university teachers, the class went to Jinan for a two-week training. Mr. Chen signed up to participate, but failed because of the weak foundation. Mr. Chen realized the importance of improving digital literacy. The failure of the training class inspired his determination to self-study. In 2015, Mr. Chen participated in a platform digital teaching ability improvement counseling class introduced by colleague, and he conducted a month long systematic training. He has learned advanced online teaching multimedia technology and is familiar with the popular online teaching organization modes. Chen has applied the learned knowledge to classroom teaching and achieved good results. In 2017, Mr. Chen school organized teacher training class, and he was hired as the keynote teacher. He shared his self-study experience in class. In 2019, Mr. Chen studied in Shanghai as a member of the training class organized by education department of government. He learned the advanced experience of online teaching in Shanghai middle schools. When talking about career planning, Mr. Chen believes that it is the key to establish the awareness of digital literacy. Although he is older, there is still much room for improvement of digital literacy.*

**Figure 2.**
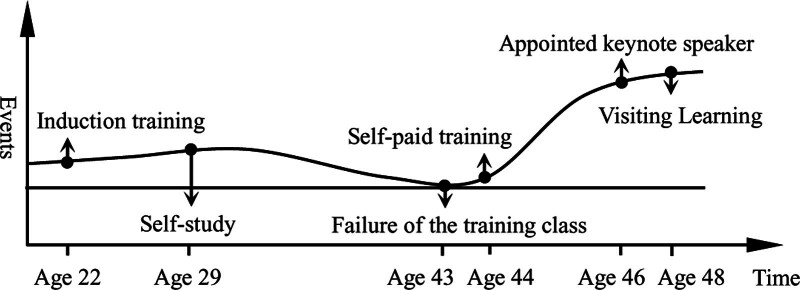
Cumulative trajectory of digital literacy of Mr. Chen.

As shown in Figure 2, the 《Fundamentals of Computer》 is an innate accumulation, and the events including self-study, self-paid training and visiting learning are continual accumulations. Under the joint influence of these 2 accumulations, the trajectory of Mr. Chen life journey has turned, especially after the self-paid training. His digital literacy has rapidly improved and teaching effectiveness has significantly improved. Mr. Chen growth story verifies that the role of continuation accumulation in the linear accumulation type is much greater than that of innate accumulation. In other words, even if medical university teachers have innate deficiencies such as professional mismatch and lack of knowledge, they can make up for them through events such as learning and training in the continual accumulation of advantage. Among the other 15 research participants, the life course events of Ms. Zhao, Mr. Feng, and Ms. Han are similar to Mr. Chen, but there is a gap between their digital literacy results due to different subjective initiative and individual cognition.

#### 4.1.2. Multi-drive accumulation

**Figure 3.**
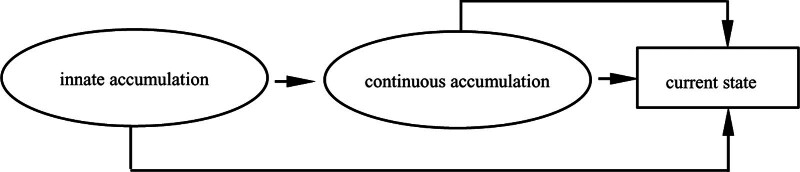
Multi-drive accumulation model.

The characteristics of multi-driven accumulation are: innate accumulation and continuous accumulation play significant roles in improving the digital literacy of medical university teachers independently. Two types accumulation influence each other and jointly contribute to the improvement of digital literacy of university teachers, as shown in Figure 3. The innate accumulation includes teachers’ cognition (forward-looking cognition of digital literacy, ability to grasp the trend of teaching change, determination to acquire skills, etc), previous accumulation (professional background, basic information skills, etc), and support from others (help from family members and colleagues, etc). The continuous accumulation includes policy guidance from educational administrative departments, teachers’ self-study, participation in training held by various organizations at all levels, gradual improvement of various online teaching platforms, etc. The accumulation includes policy guidance from the education administration, teachers’ self-learning, training held by various organizations at all levels, and the gradual improvement of various online teaching platforms.

In particular, various cumulative advantageous events occur successively in the life course of medical university teachers, which continuously enhance the cultivation effect of digital literacy and enhance the perceptual experience in the process of digital literacy enhancement. It is undeniable that cumulative disadvantageous events (e.g., encountering physical illness, etc) may occasionally occur in the career of such medical university teachers. But these disadvantageous events will not fundamentally shake the digital literacy accumulation overall career of medical university teachers, because they have accumulated many advantageous events for a long and stable period. Therefore, the multi-driven accumulation has a strong self-correcting mechanism and source of blood generation mechanism. The medical university teachers present long-term and stable characteristics in digital literacy accumulation.

*Case 2: Ms. Yang has a master’s degree and graduated from a medical university in Shandong Province. She majored in basic medicine, and minored 《Computer Basics》 《Network Technology》 《Artificial Intelligence》 etc. During her undergraduate period, she worked as an intern in an hospital for 2 years. During her master’s degree, he participated in the research work of his tutor as an important member. Ms. Yang believed that “Specialization” and “Technical ability” were key words. These indicate that “Learning experience” plays an important role in his career path. In 2013, Ms. Yang joined a medical university and became a medical university teacher in a city of Shandong Province, where the economy is relatively developed, and the conditions for running a medical university are relatively advanced. Induction training was conducted by means of self-study through online courses. She believed that professional background and internship experience played a positive role in completing the induction training. In 2018, Ms. Yang participated “Smart Campus Construction Project” of her school. Ms. Yang understood the latest technology of “Internet + Teaching.” In 2021, she won the project approval, and received 10,000 RMB of funding. She planned to pilot the construction of smart classrooms. When talking about his own experience in improving digital literacy, Ms. Yang believed that digital literacy was not only reflected in education and teaching, but also she should further strengthen her digital literacy research ability.*

**Figure 4.**
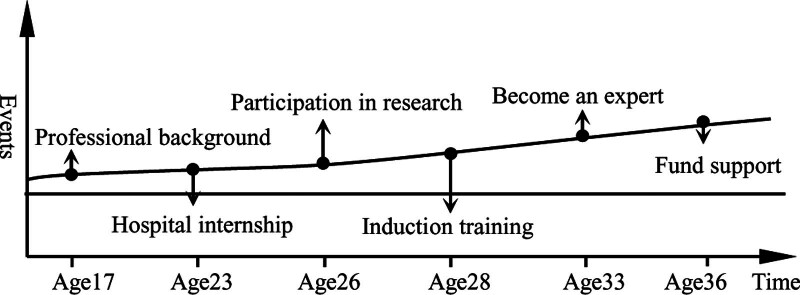
Cumulative trajectory of Digital literacy of Ms. Yang.

It can be seen from Figure 4 that the sequence of the event chain that contributed to the accumulation of Ms. Yang digital literacy is: minor courses, internship experience, participation in research, induction training, expert members, fund support. The event chain demonstrates that professional background, individual initiative, self-value orientation has played an important role in the process of the accumulation of university teachers’ digital literacy. Ms. Wang, Mr. Qian and Mr. Zheng belong to multi-drive accumulation model too. They are different from Ms. Yang in terms of professional background, internship experience and other events, but their career life trajectory after entry is generally the same. Therefore, young teachers show the characteristics of rapid change of ideas and strong ability to accept new things in the accumulation of digital literacy, while older teachers show the characteristics of rich experience and rich network resources.

#### 4.1.3. Parallel accumulation

**Figure 5.**
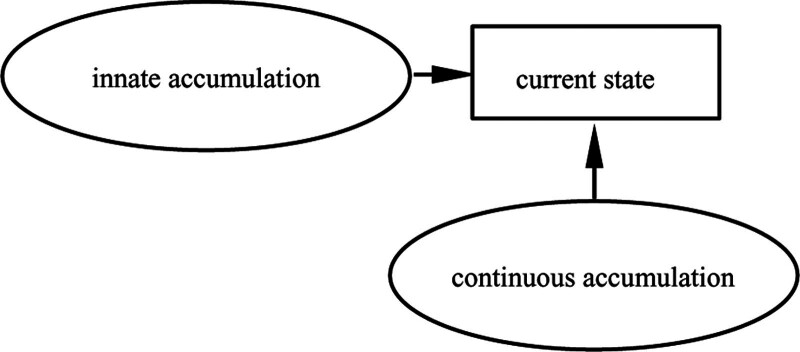
Parallel accumulation model.

Parallel accumulation is similar to linear accumulation. Innate accumulation and continuous accumulation are not effective in improving the digital literacy of medical university teachers, and they both show insufficient power in promoting accumulation advantages and counteracting accumulation disadvantages. Parallel accumulation has its significant characteristics: there is no obvious connection between innate accumulation and continuous accumulation, and they independently influence the digital literacy improvement and career trajectory of medical university teachers, as shown in Figure 5. For example, demographic factors such as age and gender in innate accumulation are not directly related to life course events such as training and self-learning in continuum accumulation. They contribute to medical university teachers’ digital literacy independently.

*Case 3: Mr. Sun graduated from a medical college in Hebei Province at the age of 21, majoring in bioengineering. During his undergraduate period, he learned 《Basic Computer Knowledge》 and had some knowledge of online learning. After graduation, Mr. Sun joined a biotechnology company of Shandong Province and was engaged in administrative work. The nature of the post made Mr. Sun have nothing to do with in-depth research on computer network technology. In 2004, as a result of caring for parents, Mr. Sun passed the examination of a medical university teacher, as a biology teacher. He has mastered certain online teaching technology through personal self-study. Gradually, Mr. Sun realized that his digital literacy could not meet the teaching needs, so he asked her colleagues. After a semester of help, Mr. Sun online teaching ability has been greatly improved. In 2019, Mr. Sun was selected by the school to participate in the training class for improving teachers’ teaching ability. The training class opened the course of 《Network Technology》 and 《Online Teaching》. His digital literacy research ability was further strengthened. In 2021, Mr. Sun made an in-depth exploration of hybrid teaching, and tried to carry out teaching reform pilot in school’s biology curriculum. The “online + offline” teaching mode strengthened the students’ learning depth.*

**Figure 6.**
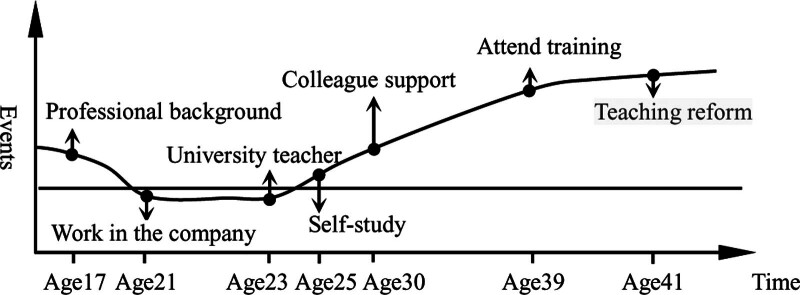
Cumulative trajectory of digital literacy of Ms. Sun.

It can be seen from Figure 6 that his innate cumulative advantages are not as obvious as Mr. Yang who has professional background. But Mr. Sun accumulated advantages of many events such as self-study, colleague support, participating in training classes, exploring teaching reform. These reflect the important role of good digital literacy accumulation in career development. Mr. Wei, Mr. Shen, and Mr. Zhou have also experienced self-study, peer guidance, teaching reform, etc, but the effect is not as good as Mr. Sun, because their subjective understanding and enthusiasm are not enough. To be sure, the digital literacy level of these 3 teachers is higher than that of other ordinary teachers.

#### 4.1.4. Lead accumulation

In the first 3 types of accumulation, the participants do not hold administrative positions, do not have managerial status, and also do not have academic positions, and the role of social accumulation in their digital literacy accumulation process is zero. In contrast, the participants in the leading accumulation type held administrative or academic positions in the school, and the participants had certain administrative and academic authority, and the social accumulation advantage played a prominent role in their digital literacy accumulation.

**Figure 7.**
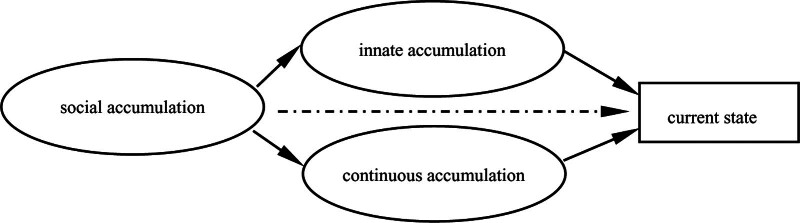
Lead accumulation model.

It was found that factors reflecting the hierarchical nature of the social division of labor, such as position, played a leading role in the process of digital literacy accumulation, and this leading accumulation both strengthened the innate accumulation advantage and contributed to the occurrence and transformation of continuation accumulation. In addition, the social accumulation due to the position held can also directly trigger the accumulation of digital literacy, as shown in Figure 7. Due to strong administrative and academic power, this type of research subject can often actively avoid negative events in their life course or reduce the cumulative disadvantage generated by negative events, while also catalyzing positive events to ensure that the cumulative advantage of positive events is maximized.

*Case 4: In 1994, Mr. Jiang went to work in his hometown’s medical university and successively served as the teaching secretary of clinical diagnosis, the leader of clinical diagnosis group, and the vice principal. Later, he obtained a master’s degree through in-service education. During his tenure as Vice President, he was responsible for the information construction and technical support of online teaching. He organized backbone teachers to learn advanced online teaching experience in other schools. As one of them, he has mastered the use of online teaching. He went out to study for half a year, learned the advanced online teaching mode of the school, and deepened his understanding of the information construction of university middle schools. In 2019, Mr. Sun upgraded the campus information system, and the school basically realized information management, especially the online teaching conditions, which not only improved the school’s information management level, but also further accumulated its own digital literacy level.*

**Figure 8.**
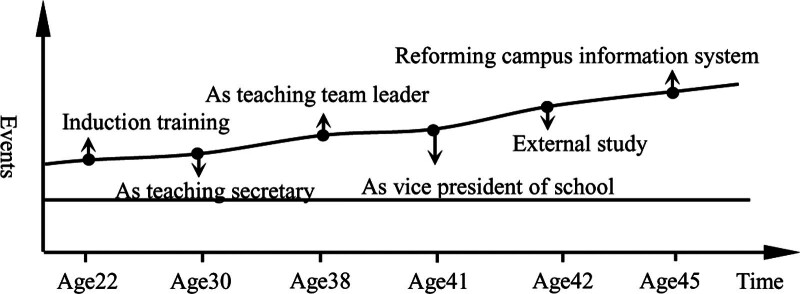
Cumulative trajectory of digital literacy of Mr. Jiang.

We can see that the process of Mr. Jiang digital literacy accumulation is basically consistent with his tenure track in Figure 8. First, his job and position required that Mr. Jiang must had a high level of digital literacy. For example, as the team leader of teaching group, he often went deep into the classroom and evaluate online teaching. Second, Mr. Jiang had the position advantage, and his political, economic, information and other powers helped him avoid the adverse effects of negative life events. Ms. Chu and Ms. Li also belong to the leading accumulation. Ms. Chu is leaders of the urology group, and Ms. Li is leaders of the gastroenterology group. But they have no experience of going out to study, and have no experience of vice principal. Therefore, their accumulated digital literacy is not as good as that of Mr. Jiang. However, they hold a positive attitude towards digitalize, actively self-study and participate in school training activities, and their digital literacy level is significantly higher than that of other ordinary teachers.

### 4.2. The role of life events

After analyzing the above types of accumulation and their characteristics, it can be learned that major life events (containing both positive and negative events) are important drivers of digital literacy accumulation among medical university teachers. Analyzing social phenomena with the life course paradigm, special attention should be paid to the length of duration between events, whether the events follow the social timeline, the sequence of major life events and their impact on future social development and the 4 types of different life course trajectories fully illustrate that the major life events of the 16 participants have different impacts on their future development, especially digital literacy accumulation and that these events continued to have an effect as they progressed.

#### 4.2.1. Peers

There are 2 stable schools of thoughts of analysis in life course theory. One is to analyze the life course of cohorts from a historical perspective, and the other is to analyze the life course of social groups from a sociocultural perspective.^[19]^ This paper analyzes the life course of medicaluniversity teachers from a historical perspective, and uses age as a stratification basis to sort out the role played by individual lives in the overall life course and the historical experiences they have undergone. When social changes act on the same generation of peer groups, the collective effects of the peer groups show the characteristics of the times. For example, the 16 participants showed that the accumulation of digital literacy was not obvious in the early stage of their life history. Due to the popularization of network information technology and the promotion of school digitalize, they all improved their digital literacy in the form of self-learning, training, and external study in the later stage of their life history. The occurrence of these events is not only an important part of individual life but also an external manifestation of the peer group effect.^[20]^

#### 4.2.2. Subjectivity

Informal control by age in life course studies is an important doctrine that individuals and social bonds are closely linked and that society controls individuals’ values and behaviors through laws and morals.^[21]^ But research found that subjective is very important in individual career development. Peers do not uniformly experience the same life events at the same time, on the contrary, the time sequence of peer experiences similar events and different events shows great difference. Research findings indicates that subjective initiative plays an important role in individual career development. For example, Mr. Sun and Mr. Wei are both belong to parallel accumulation. The former has been transferred from biotechnology companies to medical university teacher, while the latter has been transferred from administrative posts to teacher of medical university. But due to the differences in subjective understanding of digital literacy, the time and energy invested in subjective motivation are different, and there are large gaps in digital literacy accumulation, leading to different career trajectories.^[22]^

#### 4.2.3. Chronology

Chronology refers to the social timing of life events in the process of individual career development.^[23]^ Chronology is more important than the life event itself. Positive life events occurring at the most appropriate time will advance the career development process. Negative life events occurring at the least appropriate time will hinder the career development process. With efforts, individuals can control the appropriate period for life events and social roles to occur, but they are still influenced by external factors such as national policies, public social events, and physical illness. For example, Mr. Yang digital literacy accumulation event occurred at the right point in time, which contributed to his health career development. Ms. Chu met with an unexpected event, she suffered from a serious thyroid disease at the age of 36 and underwent surgery. This life event delayed her digital literacy enhancement plan, such as self-learning and attending training, which led to a change in career trajectory.

## 5. Discussion

This research analyzed and summarized the types and characteristics of digital literacy accumulation among medical university teachers and the role of life events from the perspective of life course theory, and came to the following discussions:

Firstly, the accumulation of digital literacy of medical university teachers from the perspective of life course theory can be divided into 4 types: linear accumulation, multi-driven accumulation, parallel accumulation, and leading accumulation, among which the leading accumulation type has the most obvious power of live events to promote digital literacy. From the structural model of the 4 types, it can be seen that each type includes innate accumulation and continuous accumulation, and the former shows basic stability while the latter shows directness and continuity in the individual life-course trajectory. Innate accumulation is the premise and foundation of continuing accumulation, and continuing accumulation is the articulation and continuation of innate accumulation, and the power of continuing accumulation is strengthened by the catalytic effect of innate accumulation, and the cumulative effect is generated.^[24]^ The purpose of research is to categorize the problems and phenomena of digital literacy accumulation of university teachers, find the characteristics and rules of digital literacy accumulation, and provide theoretical support and reference experience for the continuous improvement of digital literacy of medical university teachers.

Secondly, the digital literacy of medical university teachers has obvious individual differences, which is a dynamic and systematic process of accumulation of individual life events. Life is the biological attribute of teachers, digital literacy is the professional attribute of teachers, the biological attribute is the carrier of the professional attribute, and digital literacy cultivation is embedded in teachers’ career development.^[25]^ In addition, in the dynamic process of digital literacy cultivation of medical university teachers, the generation of individual life events involves multiple stakeholders, such as colleagues, schools, educational management departments, academic organizations, and online education platforms, which all play a role in different degrees in contributing to the occurrence of individual life events. Therefore, both the continuous vertical extension of teachers’ biological age and the horizontal interaction of multiple parties in teachers’ life events confirm that teachers’ digital literacy is cumulative in multiple types, and this feature is interchangeable with the teacher education concept of lifelong learning in terms of value.

Thirdly, although the Chinese government has taken various measures to improve the level of high education, but there is still a big gap in the comprehensive quality of medical university teachers compared with “Double First-class” university teachers. This is closely related to the imbalance of economic development between urban and university areas in China. The research found that educational power played a role in the accumulation of medical university teachers’ digital literacy, and teachers with higher positions can use educational power to improve their digital literacy. The accumulation of university teachers’ digital literacy is closely related to their own economic situation. High income teachers are more inclined to make greater investment in improving digital literacy.

## 6. Conclusion

In the field of Western sociological research, life course theory was first applied in the sociological study of transgressions, and later educational researchers began to explore educational events specifically in the life course, arguing that childhood is an important stage in the life course and that economic poverty in childhood will have a significant impact on education and health later in life.^[26]^

The life course theory originated in the United States, and the theory has been widely used in sociological and educational research fields and has achieved fruitful research results.^[27]^ But the theory of life course is seldom applied in the field of educational research, especially in the field of teacher education and teacher growth research. The cumulative problem of teachers’ digital literacy has been noted in relevant national policy documents on digitalize construction. 《The Education Informatization 2.0 Action Plan》 states that “Standardize the construction and application of online learning spaces, ensure that all teachers and school-age students have space for all, and carry out principal leadership and teacher application training, popularize and promote the application of online learning spaces, and realize ‘space for all’ to lead the promotion of the construction and application of online learning spaces.” The law indicates that the Chinese government has begun to focus on increasing digital literacy accumulation through training and other forms to meet the needs of online teaching.

The guidance and encouragement of policies cannot meet the needs of medical university teachers for digital literacy improvement. The medical universities urgently need a long-term and stable mechanism to accumulate digital literacy. It stimulates the occurrence of positive life events and the accumulation of advantages in the life course of medical university teachers, and promotes the transformation from the accumulated disadvantages of medical university teachers’ digital literacy to the accumulated advantages.^[28]^ The analysis of the life history trajectories of 16 medical university teachers in this research shows that subjective motivation plays an important role in the accumulation of digital literacy. The government and medical university strengthen the awareness of the cultivation of medical university teachers’ digital literacy, improve the training system of medical university teachers’ digital literacy, and build a platform for the cultivation of medical university teachers’ digital literacy, so as to constantly stimulate the emergence of positive life events, and reverse strengthen the role of subjective initiative in innate accumulation, and drive the overall improvement of medical university teachers’ digital literacy.

## 7. Limitations

The present study, while offering valuable insights into the accumulation of digital literacy among medical university teachers in China and the influence of life course events, is not without its limitations. The sample size of 16 participants, though allowing for in-depth qualitative analysis, may limit the generalizability of the findings to a broader population of medical educators or other academic settings. Future studies with larger, more diverse samples could enhance the external validity of the research. The study’s emphasis on positive life events may have overlooked the potential impact of negative or challenging events on the development of digital literacy. Understanding both positive and negative aspects would offer a more comprehensive view of the life course dynamics influencing digital literacy.

## Author contributions

**Conceptualization:** Mingling Wang, Chunguang Ling.

**Data curation:** Wei Li, Xuening Li, Xuehong Ju.

**Formal analysis:** Guangbin Ma.

**Methodology:** Shaojie Yu, Chunguang Ling.

**Software:** Shaojie Yu, Mingling Wang, Guangbin Ma.

**Validation:** Wei Li, Xuening Li, Xuehong Ju.

**Visualization:** Xuehong Ju.

**Writing – original draft:** Shaojie Yu, Chunguang Ling.
